# Preparation of the luciferase-labeled antibody for improving the detection sensitivity of viral antigen

**DOI:** 10.1186/s12985-022-01855-6

**Published:** 2022-07-28

**Authors:** Ying Tang, Yuchang Li, Sen Zhang, Jing Li, Yi Hu, Wenguang Yang, Yuehong Chen, Chengfeng Qin, Tao Jiang, Xiaoping Kang

**Affiliations:** State Key Laboratory of Pathogen and Biosecurity, The Academy of Military Medical Science. Institute of Microbiology and Epidemiology, No. 20 Dongda Street, Fengtai District, Beijing, 100071 China

**Keywords:** Nano luciferase, SARS-CoV-2, Highly sensitive, Antigen detection, Automatic magnet chemiluminescence immune assay (AMCA)

## Abstract

**Background:**

Viral antigen detection test is the most common method used to detect viruses in the field rapidly. However, due to the low sensitivity, it can only be used as an auxiliary diagnosis method for virus infection. Improving sensitivity is crucial for developing more accurate viral antigen tests. Nano luciferase (Nluc) is a sensitive reporter that has not been used in virus detection.

**Results:**

In this study, we produced an intracellularly Nluc labeled detection antibody (Nluc-ch2C5) and evaluated its ability to improve the detection sensitivity of respiratory syndrome coronavirus 2 (SARS-CoV-2) antigens. Compared with the traditional horse-radish peroxidase (HRP) labeled antibody (HRP-ch2C5), Nluc-ch2C5 was 41 times more sensitive for inactivated SARS-CoV-2 virus by sandwich chemiluminescence ELISA. Then we applied Nluc-ch2C5 to establish an automatic magnet chemiluminescence immune assay (AMCA) for the SARS-CoV-2 viral spike protein, the limit of detection was 68 pfu/reaction. The clinical sensitivity and specificity reached 75% (24/32) and 100% (48/48) using 32 PCR-positive and 48 PCR-negative swab samples for clinical evaluation, which is more sensitive than the commercial ELSA kit and colloid gold strip kit.

**Conclusions:**

Here, monoclonal antibody ch2C5 served as a model antibody and the SARS-CoV-2 served as a model pathogen. The Nluc labeled detecting antibody (Nluc-ch2C5) significantly improved the detection sensitivity of SARS-CoV-2 antigen. This labeling principle applies to other viral infections, so this labeling and test format could be expected to play an important role in detecting other virus antigens.

**Supplementary Information:**

The online version contains supplementary material available at 10.1186/s12985-022-01855-6.

## Background

Viruses cause most human infectious diseases. With the ecological and environmental changes, novel viral infectious diseases emerge continuously; viral diseases are becoming a more significant threat to health [1–3]. During the early stages of virus infection, rapid detection and accurate identification of the pathogen can determine the source of infection and infection route, thereby enabling effective prevention and control of further spread [4, 5].

Nucleic acid test such as fluorescence quantitative polymerase chain reaction (qPCR) has been the standard method for many virus identifications because of their high accuracy and high sensitivity [6–8], however, qPCR tests are complex and time-consuming, requiring special equipment, professional staff, and laboratories; thus, they cannot be used for rapid on-site/in-field detection.

The immune chromatography assay is the most common means of rapid on-site detection, which detects the virus directly in a sample; the detection process is simple and fast [4]. However, due to low sensitivity, the accuracy of detection cannot be guaranteed; thus detection of virus antigens can only be used for an auxiliary diagnosis of virus infection [9–12]. Therefore, improving detection sensitivity is essential if we are to develop more accurate and rapid viral antigen detection methods.

The sensitivity of viral antigen detection methods based on immune reactions is affected by a various factors, including the abundance of the target in the sample and the affinity of the detection antibodies for this target. Another factor is the intensity of the signal reporter [10–12]. Identifying new reporters that generate higher signal intensity, coupled with optimization of labeling strategies, may play an important role in the search for a new high- sensitivity virus antigen detection method.

The biological reporter luciferase generates a high intensity signal over a wide linear range, and has a fast enzymatic reaction. It is used widely for gene expression and gene functional analyses, as well as for in vivo and in vitro imaging. With respect to serological detection, its wider linear range, faster enzymatic reaction, and higher detection sensitivity make it a better option than other enzyme-linked reaction [13]. Nano luciferase (Nluc) is a new kind of luciferase that generates a glow-type luminescence (signal half-life > 2 h) with an activity 150-fold greater than that of either firefly (Photinus pyralis) or Renilla luciferase [13–15]. Using luciferase as reporter is expected to improve the sensitivity of virus antigen detection assays; however, no studies have reported detection of viral antigens using full-length antibodies labeled with Nluc.

SARS-CoV-2 (*genus Betacoronavirus; subfamily Ortho coronavirinae; family Coronaviridae; order Nidovirales*) contains four structural proteins: Nucleo protein (NP), spike protein (SP), envelope (E), and membrane (M) [3, 16]. In response to the pandemic, dozens of commercial SARS-CoV-2 antigen tests were developed, predominantly the lateral flow or enzyme immunoassay type. Most target NP or SP [4, 12, 17, 18]. Over the past year, we have actively explored the use of highly sensitive SARS-CoV-2-specific monoclonal antibodies (mAb) as possible detection reagents. ch2C5, a genetically engineered full-length monoclonal antibody derived from a mouse mAb binds to the spike protein receptor binding domain (S-RBD) of SARS-CoV-2 variant strains with high affinity [19], making it suitable for genetic modification and application for SARS-CoV-2 detection [14].

Here, we used mAb ch2C5 as a model antibody and the SARS-CoV-2 as model pathogen, prepared the Nluc labeled ch2C5, and examined the feasibility of using a Nluc-labeled antibody to improve the sensitivity of antigen detection. Then, a sensitive automatic magnet chemiluminescence immune assay (AMCA) for SARS-CoV-2 antigen was also developed based on Nluc-ch2C5.

## Methods

### Clinical samples, viruses, and monoclonal antibodies (Mab)

The sensitivity and specificity of the AMCA for SARS-CoV-2 was evaluated using nasopharyngeal swab (NPS) specimens collected in viral transport medium. Briefly, 32 specimens from the Central for Disease Control in Hubei province, China, were retrieved from patients with clinically suspected COVID-19 and identified as positive by SARS-CoV-2 qPCR assay. CT values ranged from 17 to 35. In addition, 48 specimens from patients with common fever were obtained from the Chinese PLA General Hospital. All tested negative by SARS-CoV-2 qPCR. All participants provided written informed consent, and this study was approved by the ethics committee of the Academy of Military Medical Science. All specimens were stored at −70 °C until required.

Supernatants from SARS-CoV-2 BetaCoV/Beijing/IME-BJ05/2020 cultures (8 × 10^6^ pfu/ml) were collected and inactivated using ß-propiolactone for the sensitivity assay. Specificity was determined by testing heat-inactivated culture supernatants from nine species of respiratory tract infection-associated virus: adenovirus types 3(ADV3); influenza A viruses H1N1, H3N2, and H7N9; influenza B virus (INFB); para influenza viruses 1 and 2(PIF1 and PIF2); rubella virus (RV); and respiratory syncytial virus (RSV).

MAb MW06, a specific antibody targeting the spike protein of SARS-CoV-2 [20], as denoted by Mabwell Inc. (Shanghai, China). ch2C5, a recombinant engineered full-length chimerical mAb derived from the murine hybridoma cell line 2C5, targets the S-RBD of SARS-CoV-2 with high sensitivity [21]. This mAb comprises the constant region of human antibody IgG1 plus the variable region of the murine antibody 2C5.

### Construction of recombinant expression plasmids for Nluc-ch2C5

First, pcDNA3.1 (Promega, USA) was used to construct the light chain expression vector pcDNA3.1-ch2C5L, and the heavy chain expression vector pcDNA3.1-ch2C5H-Nluc (Nluc was fused to the end of the antibody heavy chain). To construct pcDNA3.1-ch2C5L, an 810 bp gene fragment comprising the signal peptide and the ch2C5 light chain was generated by overlapping PCR and then inserted into pcDNA3.1 between the AflII and EcoRI cloning sites.

**Fig. 1:**
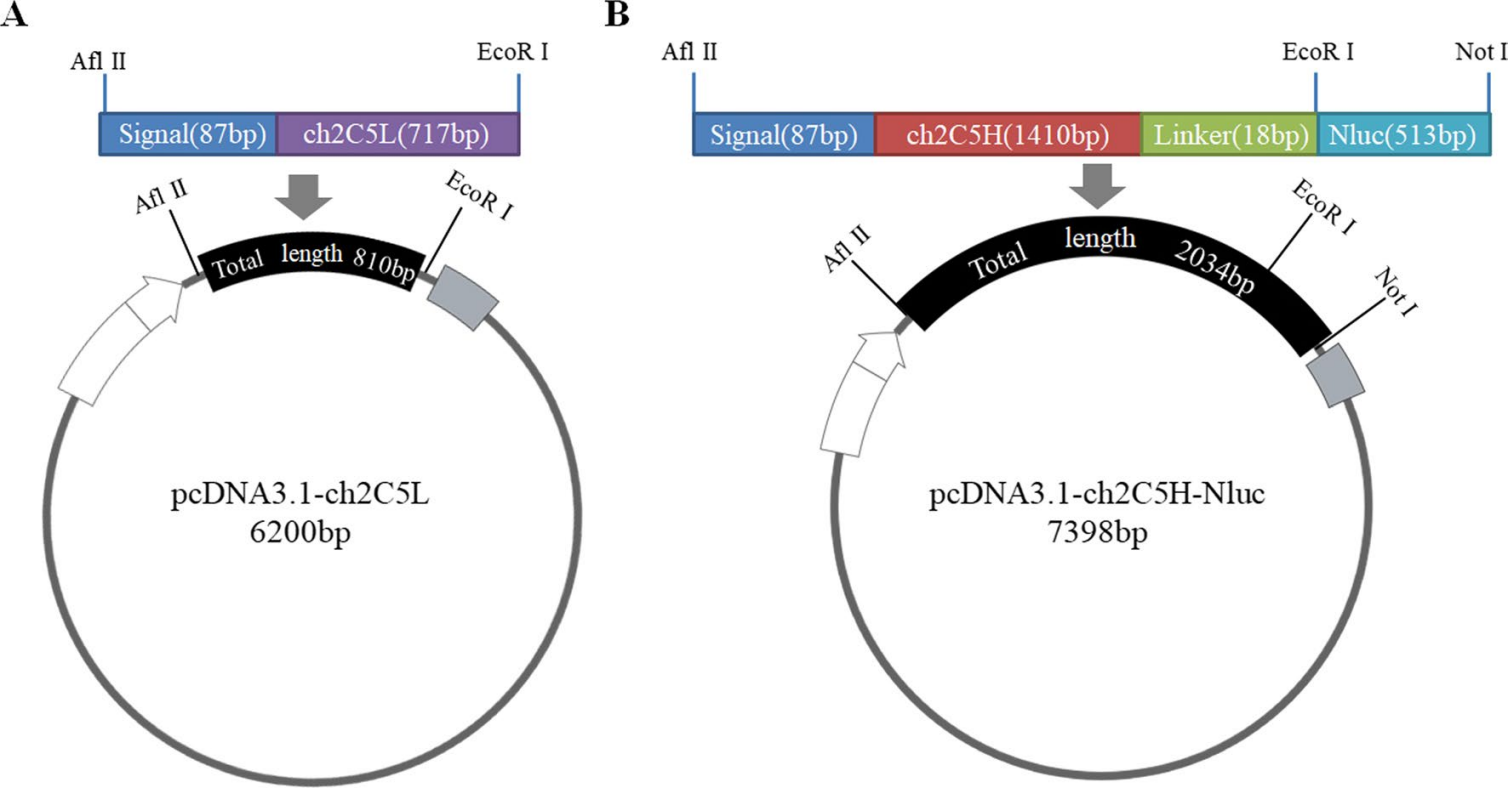
Maps of the vectors used for Nluc-ch2C5 production. **A** The pcDNA3.1-ch2C5L vector. The 810 bp fragment comprising a signal peptide sequence and the ch2C5 light chain was created by overlapping PCR and then inserted into pcDNA3.1 between the AflII and EcoRI sites to produce pcDNA3.1-ch2C5L. **B** pcDNA3.1-ch2C5H-Nluc. The 2034 bp gene fragment comprising the signal peptide, the ch2C5 heavy chain, an 18-mer linker, and Nluc was inserted into pcDNA3.1.

To construct pcDNA3.1-ch2C5H-Nluc, a 1503 bp gene fragment comprising the signal peptide and ch2C5 heavy chain was generated by overlapping PCR and then inserted into pcDNA3.1 between the AflII and EcoRI cloning sites to yield pcDNA3.1-ch2C5H. To ensure the flexibility required to maintain antigen binding capability, an 18-mer linker was fused with Nluc by overlapping PCR (final fragment size of 531 bp) and inserted into the pcDNA3.1-ch2C5H vector between the EcoRI and Not I sites to yield the pcDNA3.1-ch2C5H-Nluc vector. A total of 2034 bp fragment was inserted. The maps of pcDNA3.1-ch2C5L and pcDNA3.1-ch2C5H-Nluc are shown in Fig. 1.

### Expression and purification of the Nluc-ch2C5 antibody

Nluc-ch2C5 was produced by transient transfection with a mixture of pcDNA3.1-ch2C5L and pcDNA3.1-ch2C5H-Nluc. In brief, COS-7 cells in Dulbecco’s modified Eagle’s medium (Gibco, USA) supplemented with 10% fetal bovine serum (Gibco, USA) were cultured in 24-well plates in a humidified 5% CO_2_ incubator at 37 °C. When the cells reached 80% confluence, they were co-transfected with pcDNA3.1-ch2C5L and pcDNA3.1-ch2C5H-Nluc at a ratio of 1:1 (0.25 μg/0.5 μg) using Lipofectamine 3000 transfection reagent (Invitrogen, USA). The cell culture supernatant was collected at 24, 48, 72, and 96 h post-transfection.

To measure luciferase luminescence intensity, 50 μl of cell culture supernatant was mixed with 50 µl of NLuc substrate (Promega, USA) and placed immediately in a GloMax Multi Microplate Reader (Promega, Madison, WI, USA) for luminescence analysis. The Nluc-ch2C5 antibody was purified using protein A/G PLUS agarose (Santa Cruz, CA) and subjected to 12.5% sodium dodecyl sulfate–polyacrylamide gel electrophoresis (SDS-PAGE) and Coomassie Brilliant Blue R250 staining/luciferase luminescence imaging to identify the antibody band.

### Preparation of HRP-ch2C5

Monoclonal antibody ch2C5 was coupled to HRP using an Ez-link™ Maleimide Activated HRP kit (Pierce, USA). The labeled antibody (HRP-ch2C5) was stored in small aliquots in a −70 °C freezer.

### Measurement of antibody binding kinetics by biolayer interferometry (BLI)

The affinity assay was conducted at room temperature using a Gator™ Label-Free Bioanalysis system (Gator Bio, Palo Alto, CA, USA). To measure the affinity of the labeled antibodies for the S-RBD protein, the latter was diluted to 796 nM in Q Buffer (PBS [pH 7.4], 0.02% Tween 20, 0.2% bovine serum albumin (BSA), and 0.05% NaN_3_) and captured by an anti-His-tag sensing probe chip (GE Healthcare). The blank channel of the chip served as the negative control. Nluc-ch2C5 or HRP-ch2C5 was serially diluted twofold (from 118 to 1.88 nM) with Q buffer and loaded onto the sensing probes. After each cycle, the sensor was regenerated with Gly-HCl (pH 1.5). Affinity was calculated by Gator evaluation software using a 1:1 (Rmax Local fit) binding fit model and expressed as an affinity constant [21].

### Antigen-binding activity of Nluc-ch2C5 in a direct ELISA

To test the ability of Nluc-ch2C5 to bind to the S-RBD of SARS-CoV-2, an antigen-binding activity assay was conducted in a mode similar to that of a direct ELISA; the S-RBD protein (Sino Biological, China) was used as the antigen, and BSA, the recombinant envelope glycoprotein of Japanese encephalitis virus, and the recombinant envelope glycoprotein of tick-borne encephalitis virus were used as negative controls.

To measure the sensitivity of Nluc-ch2C5 for S-RBD, S-RBD was serially diluted fourfold (1 μg/ml–0.06 ng/ml) and used as the detection antigen. S-RBD and negative controls were coated (in triplicate) overnight at 4 °C onto white 96-well polystyrene plates (Costar, USA). After blocking with PBST (phosphate-buffered saline/0.05% Tween-20) containing 3% BSA, the Nluc-ch2C5 antibody (10 ng/ml, 100µl/well) was added to the wells and incubated for 1.5 h at 37 °C. After washing, 100 μl of Nano luciferase substrate (Promega, USA) was added to each well and luminescence intensity was measured by a GloMax microplate luminescence detector.

The average value of the negative control group plus three standard deviations was set as the cutoff value; samples higher than the cutoff value were deemed positive. The ratio of the average luminescence intensity of each test sample to the cutoff value (S/C) was calculated. An S/C value > 1 was taken as the positive threshold for results analysis.

### Antigen-binding activity of Nluc-ch2C5 in a double-antibody sandwich ELISA

Next, a double-antibody sandwich ELISA was performed to determine the sensitivity of the Nluc-ch2C5 antibody for SARS-CoV-2 or S-RBD. The capture antibody MW06 (2 μg/ml) was coated overnight at 4 °C onto a white 96-well plate. After blocking with PBST/3% BSA, inactivated SARS-CoV-2 (10^5^pfu/ml–39pfu/ml, serially diluted five-fold) or S-RBD (1 μg/ml–0.06 ng/ml, serially diluted four-fold) was added to the wells for 1.5 h at 37 °C. Inactivated H7N9 and 3% BSA were used as negative controls, and 0.1% BAS-PBST was used as a blank control. After washing, Nluc-ch2C5 (10 ng/ml) was added to the wells at 37 °C for 1.5 h. After washing five times with PBST, 100 μl of Nano luciferase substrate was added. The average value of the negative control group plus three standard deviations was set as the cutoff value; samples showing values higher than the cutoff value were deemed positive. The ratio of the average luminescence intensity in each test sample to the cutoff value (S/C) was calculated. An S/C value > 1 was taken as a positive threshold for results analysis.

### Antigen-binding activity of HRP-ch2C5

Using HRP-ch2C5 antibody (10 ng/ml, 100µl/well) as the detecting antibody and catalyzed with chemical luminescence substrate (CWBIO Technology, Beijing, China), the sensitivity of HRP-ch2C5 was determined using the direct ELISA and the double-antibody sandwich ELISA assay, the protocols were similar as mentioned above.

### AMCA

**Fig. 2:**
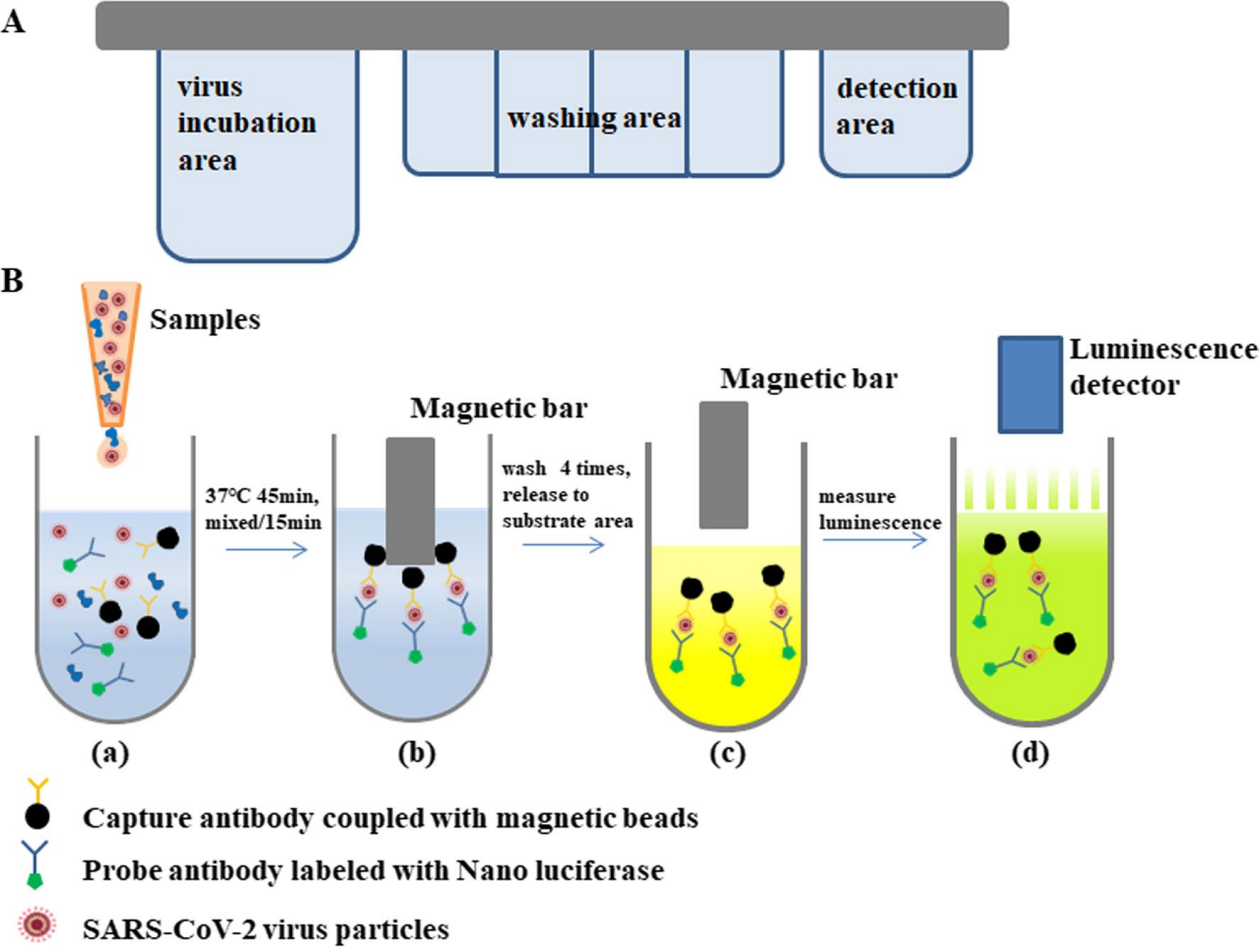
**A** Schematic diagram of the cartridge, which comprises three areas: a virus incubation area, a washing area, and a detection area. **B** Schematic illustration of the AMCA. (a) 200 μl of sample, 5 μl of magnetic beads (10 mg/ml), and 150 μl of Nluc-ch2C5 (10 ng/ml) were mixed in virus incubation area and incubated for 45 min at 37℃. The mixture is agitated every 15 min. (b) The magnetic bar pulls the magnetic beads into the washing area and the beads are washed four rounds. (c) The magnetic beads are released into the detection area (Nano-Glo substrate). (d) Luciferase intensity is measured

The AMCA device comprises a viral antigen detection cartridge and an automatic magnetic particle detector (BGI Inc, China). The cartridge has three areas: a virus incubation area, a washing area, and a detection area (Fig. 2A). In this system, magnetic beads are utilized as the viral antigen-capture material, which provides a large specific surface area that can bind more target antigens than a traditional flat plate; also, the beads can be removed controllably, easily, and rapidly by the magnet.

The magnetic bar in the automatic magnetic particle detector pulls the magnetic beads into the virus incubation area for viral antigen capture, into the washing area for washing out of nonspecific proteins, and into the detection area to measure luminescence intensity. A schematic illustration of the AMCA is shown in Fig. 2B.

### Preparation of magnetic bead-antibody complex for capture of SARS-CoV-2

The beads were coupled to the antibody in accordance with the manufacturer’s instructions. The following steps were performed: 2 mg of Magnosphere™ MS300/Carboxyl (JSR Life Science, Japan) was washed three times with 2-morpholine-ethane sulfonic acid buffer (MES buffer; 0.015 M, pH 5.5), followed by the addition of 20 µl of 1-Ethyl-[3,3-dimethyl-aminopropyl] carbodiimide (EDC; 10 mg/mL) and 20 µl of N-hydroxy succinimide (NHS;10 mg/ml) to 200 µl of magnetic beads. Mixing was performed in a vertical mixer for 30 min at 37 °C. The magnetic beads were washed and re-suspended in MES buffer, and 40 μg of antibody was added. Mixing was performed using a vertical mixer for 4 h to allow coupling. After that, the magnetic beads were separated using a magnet and washed twice with PBST. Finally, the coupled beads were collected and stored at 4 °C in 200 μl of 3% BSA until further use.

### AMCA detection procedure

Sample (200 μl), magnetic beads (5 μl;10 mg/ml), and Nluc-ch2C5 (150 μl; 10 ng/ml) were added to the virus incubation area of the cartridge and incubated at 37 °C for 45 min; the mixture was agitated automatically every 15 min using a pipette tip. Next, the magnetic bar in the device pulled the magnetic beads into the washing areas for four rounds of washing. The luminescence intensity was measured after the magnetic beads were pulled and released into the detection area.

### Analytical sensitivity of AMCA

To assess analytical sensitivity, LODs were calculated using SARS-CoV-2 BetaCoV/Beijing/IMEBJ05/2020 strain. First, preliminary dilution experiments were done (tenfold diluted SARS-CoV-2 BetaCoV/Beijing/IMEBJ05/2020 strain; 10^6^pfu/ml–10pfu/ml).

Next, three individual replicate reactions and eight replicates per sample per experiment were performed using concentrations around the detection end point determined in the preliminary dilution experiments. The virus culture was serially diluted twofold from 2000 pfu/ml–62.5pfu/ml), and 200 µl aliquots were used to evaluate the sensitivity of the AMCA.

### Statistical analysis

The LODs were calculated by Probit analysis. The 95% confidence interval (CI) was calculated using a Probit regression model in SPSS statistical software (version 19.0; IMB).

For clinical sensitivity and specificity analysis, the average value of the negative control group plus three standard deviations was set as the cutoff value, the ratio of the average luminescence intensity in each test sample to the cutoff value (S/C) was calculated. An S/C value > 1 was taken as a positive threshold for results analysis. One way ANOVA tests were used to compare the results for SARS-CoV-2 infected cases and uninfected cases. *P* value of < 0.05 was deemed significant. ROC curves were constructed to calculate the sensitivity and specificity of the AMCA.

## Results

### Preparation and characterization of the Nluc-ch2C5 antibody

**Fig. 3:**
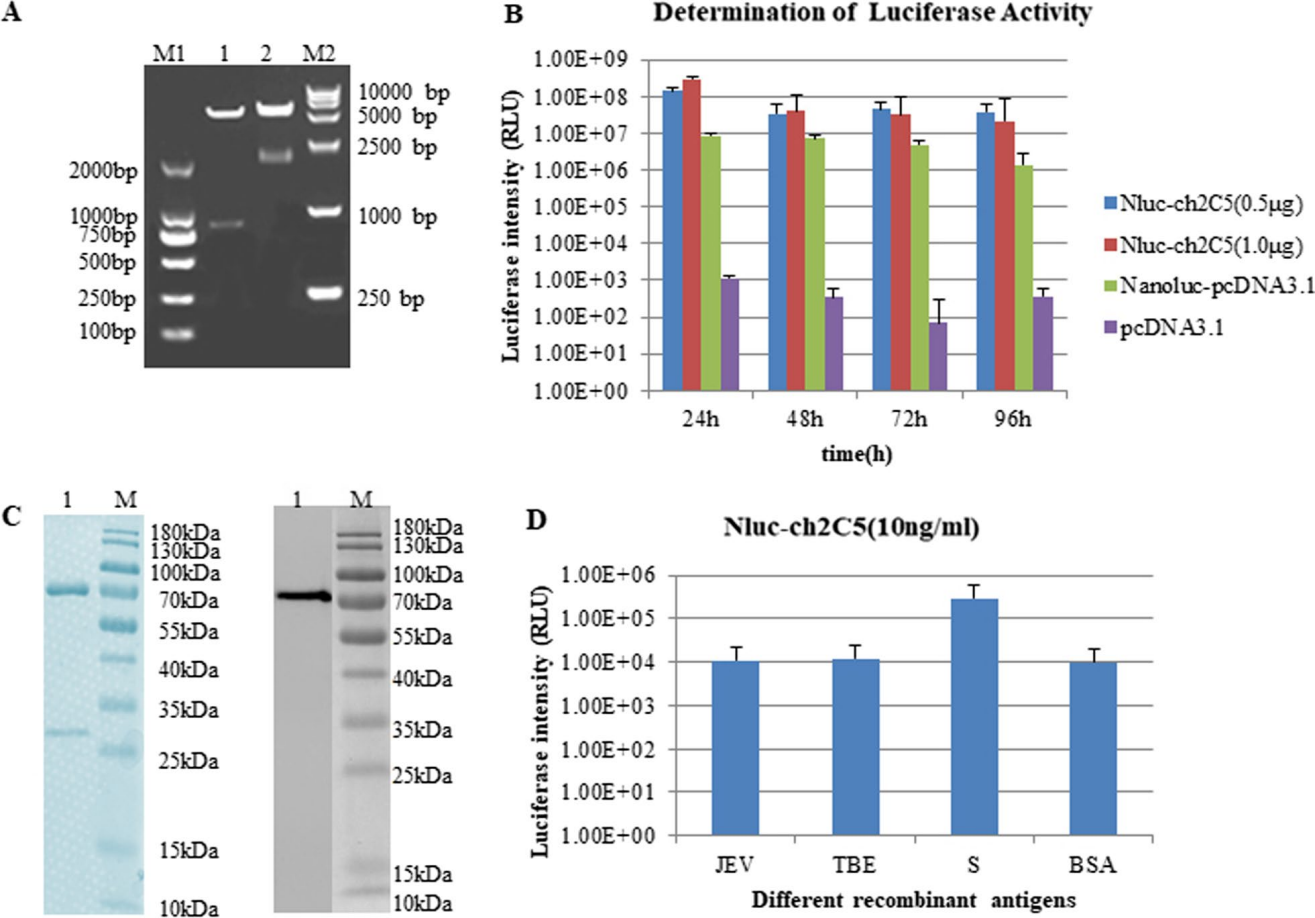
Preparation and characterization of the Nluc-ch2C5 antibody. **A** Analysis of recombinant plasmids by restriction endonuclease digestion. Lane 1, plasmid pcDNA3.1-ch2C5L digested with AflII and EcoRI. The inserted gene fragment, comprising the signal peptide and the ch2C5 light chain, is about 810 bp. Lane 2, plasmid pcDNA3.1-ch2C5H-Nluc digested by the same restriction endonucleases. The inserted gene fragment is about 2034 bp (signal peptide, ch2C5 heavy chain, linker, and Nluc). **B** Detection of luciferase activity in culture supernatant over 4 consecutive days. The light and heavy chain plasmids were co-transfected (at a 1:1 ratio) into COS-7 cells. Nanoluc-pcDNA3.1 (containing the Nluc gene only) and pcDNA3.1were used as negative controls. **C** Polyacrylamide gel electrophoresis of the purified Nluc-ch2C5 antibody. left: Coomassie blue staining; right: luciferase luminescence imaging analysis. **D** Binding of Nluc-ch2C5 to S-RBD.

Two recombinant plasmids, pcDNA3.1-ch2C5L and pcDNA3.1-ch2C5H-Nluc (Fig. 1), were constructed to allow expression of Nluc-ch2C5. Restriction endonuclease digestion and gene sequencing confirmed that the size of fragments was as expected: 810 bp and 2034 bp (Fig. 3A).

After co-transfecting the two recombinant plasmids into COS7 cells, cell culture supernatant was collected, and every 24 h to measure luciferase luminescence intensity. Intensity reached 10^8^ 24 h after plasmid transfection, indicating a high level of Nluc-fused antibody expression (Fig. 3B). The supernatant was collected and Nluc-ch2C5 was isolated using protein A/G magnetic beads prior to SDS-PAGE. Protein Bands of ~ 70 kDa and ~ 25 kDa are visible on the Coomassie-stained gel (Fig. 3C (left)), which are the predicted masses of the Nluc-fused heavy chain and light chains, respectively. Luciferase luminescence imaging analysis revealed a bright band of 70 kDa, reflecting the luciferase luminescence activity of Nluc-ch2C5 heavy chain (Fig. 3C right).

Next, we used a direct ELISA to detect binding of Nluc-ch2C5 to the S-RBD protein of SARS-CoV-2. The results are shown in Fig. 3D. The luminescence intensity in the test group was about tenfold higher than that in the control group, indicating that the recombinant Nluc-ch2C5 antibody retained specific binding affinity for the S-RBD protein.

### The affinity of the Nluc-ch2C5 antibody for the S-RBD protein is slightly higher than that of the HRP-ch2C5 antibody

**Fig. 4:**
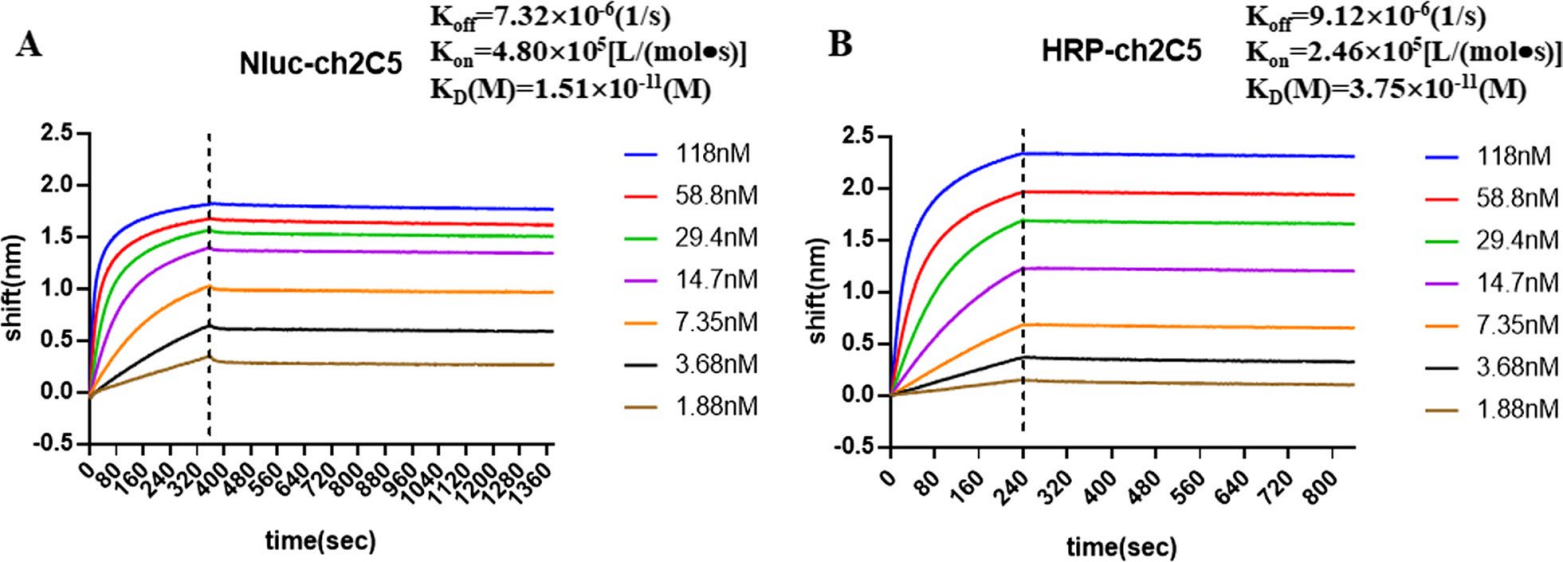
Affinity measurement. **A**: Affinity of Nluc-ch2C5 for S-RBD. **B**: Affinity of HRP-ch2C5 for S-RBD. The affinity and dissociation constant were calculated by Gator evaluation software using a 1:1 (Rmax Local fit) binding model

Biolayer interferometry (BLI) assay was used to compare the affinity of Nluc-ch2C5 and HRP-ch2C5 for the S-RBD protein. The results showed that the dissociation constant (K_D_) for binding of Nluc-ch2C5 and HRP-ch2C5 to S-RBD was 1.51 × 10^–11^ M and 3.75 × 10^–11^ M, respectively (Fig. 4), suggesting that the binding affinity of Nluc-ch2C5 antibody to S-RBD is twice as that of HRP-ch2C5.

### Nluc-ch2C5 is significantly more sensitive than HRP-ch2C5 in a double-antibody sandwich ELISA

**Fig. 5:**
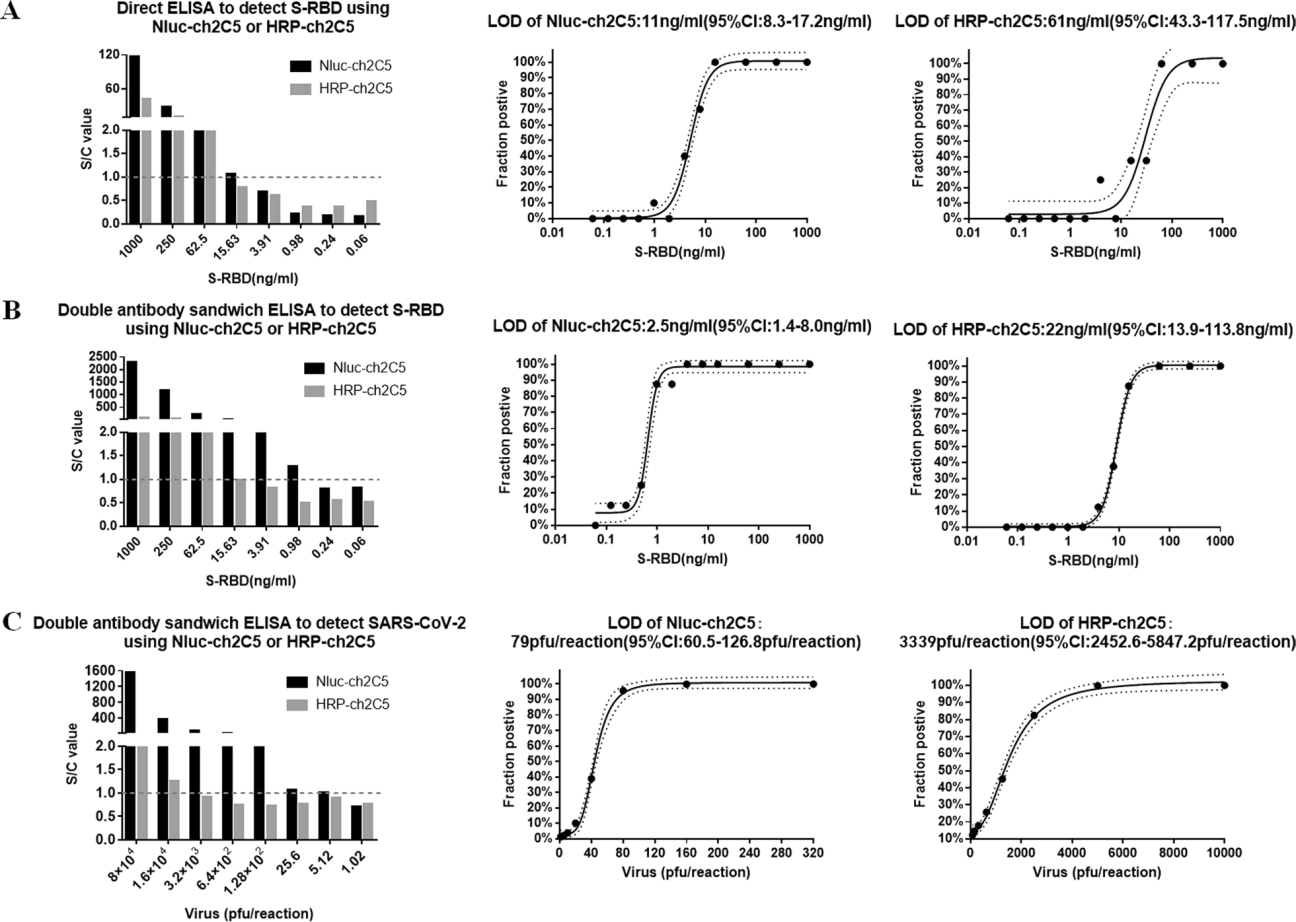
LODs of Nluc-ch2C5 and HRP-ch2C5. **A**: LODs of Nluc-ch2C5 and HRP-ch2C5 for S-RBD in the indirect ELISA. The S/C values (left); LOD of Nluc-ch2C5 (middle); LOD of HRP-ch2C5(right). **B**: LODs of Nluc-ch2C5 and HRP-ch2C5 for S-RBD in the double-antibody sandwich assay. S/C values (left); LOD of Nluc-ch2C5(middle); LOD of HRP-ch2C5(right). **C**: LODs of Nluc-ch2C5 and HRP-ch2C5 for SARS-CoV-2 in the double-antibody sandwich assay. S/C values (left); LOD of Nluc-ch2C5 (middle); LOD of HRP-ch2C5 (right). S/C is the value of the average luminance intensity for the test group divided by the cutoff value (average luminance intensity plus three standard deviations). The dotted line denotes S/C = 1 (in left figures). The inner line is a Probit curve (does-response rule) and the outer dotted/dashed lines are the 95% confidence intervals(in middle and right figures)

Using serially diluted S-RBD protein as the test antigen, the sensitivities of Nluc-ch2C5 and HRP-ch2C5 were tested in a direct ELISA first. Three independent experiments, each with eight different S-RBD concentrations, were conducted. For results analysis, the average luminescence intensity of the control group plus three standard deviations were set as the cutoff value. The ratio of the average luminescence intensity of each test sample to the cutoff value (S/C) is shown in Fig. 5A. Samples for which S/C > 1 showed positive for S-RBD binding. Probit analysis was used to calculate the LOD for both Nluc-ch2C5 and HRP-ch2C5. The LOD reached for Nluc-ch2C5 and HRP-ch2C5 was 11 ng/ml and 61 ng/ml, respectively, meaning that the sensitivity of Nluc-ch2C5 for S-RBD was 4.5-fold higher than that of HRP-ch2C5 (Fig. 5B).

Next, we calculated the LOD of Nluc-ch2C5 and HRP-ch2C5 in a double-antibody sandwich ELISA; this is most common format used by viral antigen detection kits since it can enrich the virus particles from the sample using a capture antibody, making the assay more sensitive than the direct antigen assay.

The LOD of Nluc-ch2C5 and HRP-ch2C5 for SARS-CoV-2 S-RBD was 2.5 ng/ml and 22 ng/ml, respectively (Fig. 5B), suggesting that Nluc-ch2C5 is about eightfold more sensitive than HRP-ch2C5 for S-RBD. Regarding detection of whole inactivated virus, the LOD for Nluc-ch2C5 and HRP-ch2C5 was 79 pfu/reaction and 3339 pfu/reaction, respectively (Fig. 5C), suggesting that Nluc-ch2C5 is about 41 times more sensitive than HRP-ch2C5.

### Sensitivity and specificity of the AMCA for SRAS-CoV-2

**Fig. 6:**
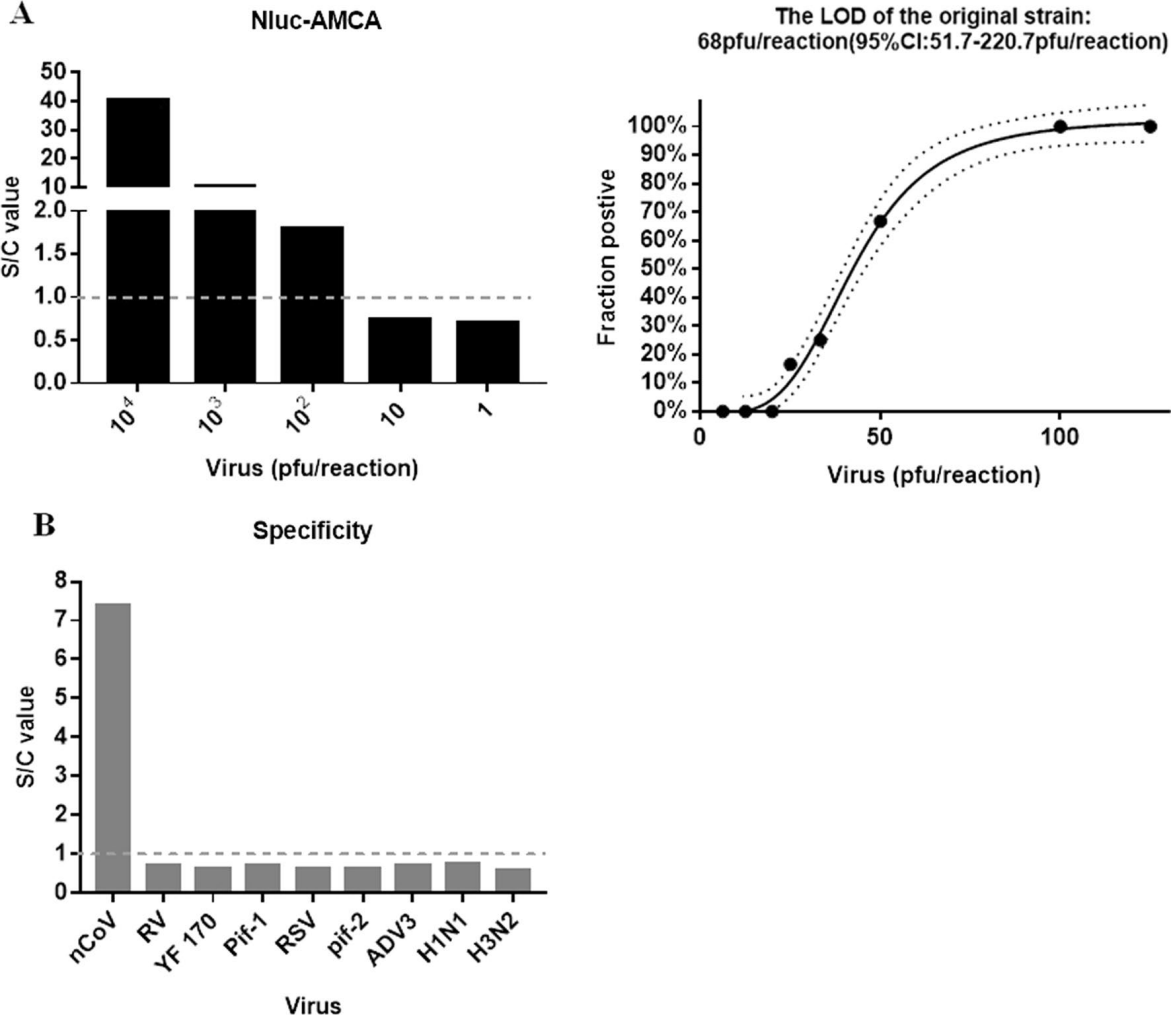
**A** Sensitivity and specificity of Nluc-AMCA for SARS-CoV-2. Sensitivity of the Nluc-AMCA for SARS-CoV-2. Left: S/C value. Right: The LOD of AMCA for SARS-CoV-2. The x-axis shows the amount of virus in each reaction (pfu/reaction). The y-axis shows the percentage of positives in all parallel reactions performed. The solid black line is the Probit curve (dose–response). The outer dotted/dashed lines are the 95% confidence intervals. **B** Specificity of the AMCA for SARS-CoV-2.

The schematic illustration of AMCA is shown in Fig. 2. To assess analytical sensitivity, LOD was calculated using SARS-CoV-2 BetaCoV/Beijing/IMEBJ05/2020 strain. Preliminary dilution shows that the LOD is about 100 pfu/reaction (1000pfu/ml). Next, the virus culture was twofold serially diluted to evaluate the sensitivity of the AMCA. The results revealed that the LOD from replicate tests was 68 pfu/reaction (95% CI: 51.7–220.7), as shown in Fig. 6A.

Next, we used virus cultures including influenza A virus H1N1\H3N2\H7N9, influenza B virus, PIV1 and PIV2), RV, InfB, and ADV3 as antigens to confirm the specificity of the AMCA. There was no cross-reaction between any of these viruses and SARS-CoV-2 (Fig. 6B).

### Validation of the AMCA using clinical samples

Validation of the AMCA was conducted using 32 qPCR-positive NPS specimens and 48 qPCR-negative specimens. The AMCA results were compared with those of commercial ELISA kits and a colloid gold strip for SARS-CoV-2 detection.

**Fig. 7:**
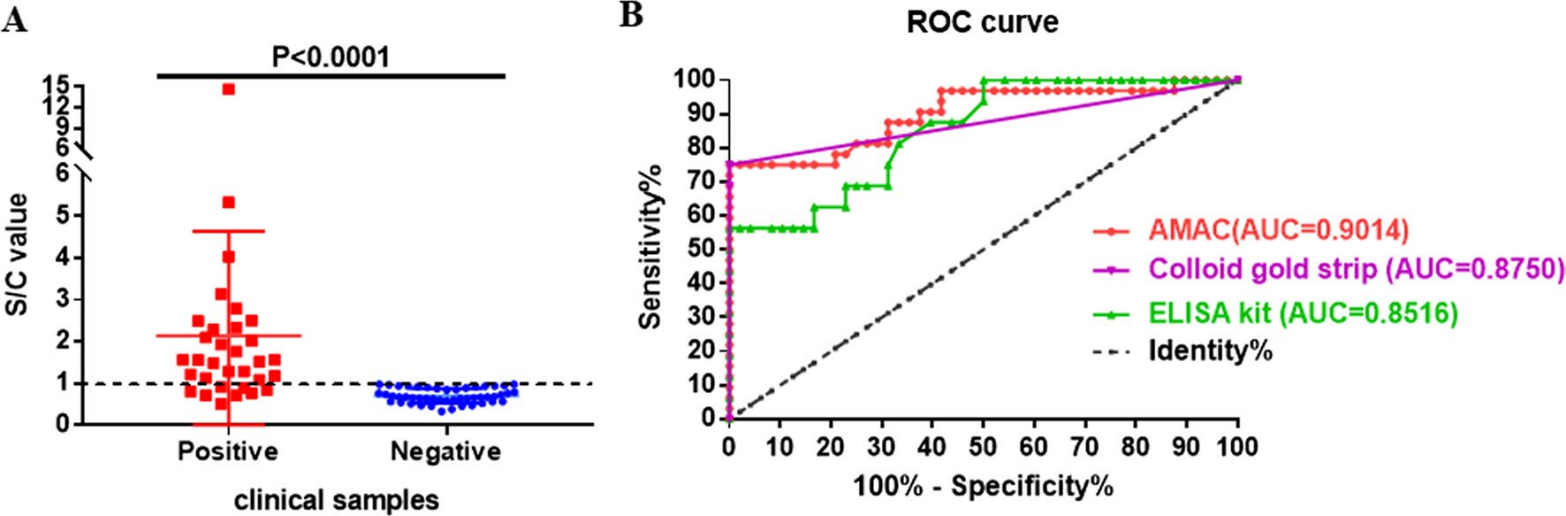
Clinical Sensitivity and specificity comparison of the Nluc-AMCA with commercial ELISA and colloid gold strip kit. **A**: Scatter diagram of Nluc-AMCA; **B**: ROC curve of the Nluc-AMCA, commercial ELISA and colloid gold strip. SARS-CoV-2 negative NPS (n = 48) were applied to Nluc-AMCA to assess specificity and 32 positive NPS were used to test sensitivity. All of the samples were identified by qPCR. The clinical sensitivity and specificity of the Nluc-AMCA were reached 75% and 100% respectively. AUC for the Nluc-AMCA, commercial ELISA and colloid gold strip is 0.9014, 0.8750, 0.8516, respectively

The AMCA results are shown in Fig. 7A, and a comparative of AMCA with commercial kits is shown in Fig. 7B and the Additional file 1: Table. The AMCA correctly identified 24 positive and all negative samples, meaning that the specificity was 100% and the sensitivity was 75%, the accuracy is 90.14%. All 24 positive samples had a CT value < 31 in the diagnostic qPCR, while the eight negative samples had a CT value of > 31 cycles. Comparing the clinical sensitivity result with that of a commercial ELSIA kit and a colloid gold strip, both produced by WANTAI BioPharm (Beijing, China) revealed that both showed the eight false-negative samples in AMCA as true negatives. The ELISA kit identified 20 of the 24 AMCA-positive samples and the colloid gold strip identified 22, revealing a clinical sensitivity of 62.5% and 68.75%, respectively; thus, the AMCA was more sensitive than the ELISA and the colloid gold strip.

## Discussion

At present, qPCR is the most widely used and accurate method for detecting SARS-CoV-2. However, antigen detection tests that are not as labor-intensive are also increasingly being deployed, although their sensitivity is low. As such, they can be used only as a supplementary method for detecting virus infection [4, 12].

Most detecting antibodies used in viral antigen detection methods such as the colloidal gold method or chemiluminescence ELISA are labeled with a reporter (e.g., HRP, biotin, or fluorescein) using chemical methods in which the reporter is usually conjugated to a primary amine or carboxyl group of an amino acid residues located in the antibody sequence. However, amino acid residues locating in the antibody variable region are often involved in protein–protein interactions necessary for protein function; therefore, reporters may interfere with function and reduce antibody affinity and detection sensitivity. For mAbs in particular, modification of a critical amino acid residue will impair all antibody molecules because they are identical [22–24]. Thus, much care is needed when developing labeling protocols for mAbs [25, 26]. Gene recombination technology can be used to ensure that the reporter is attached to specific position away from the antigen recognition site, thus the reporter is less likely to interfere with antigen binding [27–30].

In this study, we used mAb ch2C5 as the model detecting antibody and Nano luciferase as the reporter; the aim was to improve sensitivity for SARS-CoV-2 antigen. Nano luciferase is a sensitive reporter because (1) it can generate stronger signals than traditional reporters such as HRP; (2) labeled antibodies retain their activity. Here, we used genetic engineering technology to label the antibody with the Nluc reporter, ensuring that it did not interfere with antigen binding or expression of the antibody in cell culture; and (3) the labeling process is simple. Cell culture supernatant containing the labeled antibody can be used directly for virus antigen detection without purification, thereby avoiding loss of activity during the purification process.

In addition to the advantages mentioned above, the molecular weight of Nluc is only 19KD; due to the absence of disulfide bonds, glycosylation, and other modifications, Nluc has a high intracellular expression yield, a stable structure, and shows high tolerance to changes in temperature and pH [13, 14]. All of these properties make it suitable for intracellular labeling to generate a more sensitive and stable reporter for virus antigen detection.

We constructed a recombinant Nluc-labeled antibody expression plasmid and expressed Nluc-ch2C5 in cultured cells. To evaluate the effect of Nluc-ch2C5 antibody in improving the sensitivity of SARS-CoV-2 antigen detection, we prepared the HRP-labeled ch-2C5 (HRP-ch2C5) and compared its affinity and sensitivity for SARS-CoV-2 antigen with that of Nluc-ch2C5.

The affinity of Nluc-ch2C5 was slightly higher than that of HRP-ch2C5, and a direct ELISA showed that it was 4.5 times more sensitive for S-RBD than HRP-ch2C5. This may be due to the stronger luminescence signal generated by Nluc.

Most clinically available virus antigen detection reagents use the double-antibody sandwich detection mode [4, 10, 11], which uses one antibody to capture the virus antigen from the sample and a labeled antibody to bind to the captured virus antigen and produce a signal. Because the capture step enriches the virus antigen, the sensitivity of the sandwich ELISA is significantly higher than that of the direct ELISA.

We evaluated the sensitivity of Nluc-ch2C5 in a double-antibody sandwich assay. In this assay, both Nluc-ch2C5 and HRR-ch2C5 showed higher sensitivity than in the direct ELISA. The LOD of Nluc-ch2C5 and HRR-ch2C5 for S-RBD was 2.5 ng/ml and 22 ng/ml, respectively, in the double-antibody sandwich assay, making the LOD of Nluc-ch2C5 eight times lower than that of HRP-ch2C5. When inactivated SARS-CoV-2 was used as the antigen, the LOD of Nluc-ch2C5 was 41 times lower than that of HRP-ch2C5. In addition to the higher signal intensity of Nluc, it is speculated that the main reason for the higher sensitivity of Nlu-ch2C5 than HRP-ch2C5 in the double-antibody sandwich assay is the intracellular labeling method. The Nluc reporter was added to the end of the heavy chain constant region, away from the antigen binding site, resulting in lower steric hindrance than is the case for HRP-ch2C5, thus this kind of labeled antibody is more conducive to antigen binding in the double-antibody sandwich mode.

Nluc-ch2C5 and HRP-ch2C5, two antibodies with an identical protein sequence but labeled with a different reporter, show very different sensitivities. In addition to ch2C5 antibody, we also prepared luciferase labeled antibody Nluc-ch2B5 for tick-borne encephalitis virus detection using the same method. Compared with the traditional HRP labeled ch2B5 antibody, the Nluc-ch2B5 also improved the detection sensitivity significantly, with the LOD decreased about 30 times than that of HRP-ch2B5(data not shown). These suggest that intracellular labeling with Nluc would be valuable for developing a more sensitive viral antigen detection method.

However, the genetic method for Nluc labeling has the drawbacks such as being time- consuming and the need for the variable sequence of the antibody, in many cases, gene sequences cannot be obtained for the antibodies, such as commercially purchased antibodies, which can’t be labeled by genetic methods. To evaluate the availability of the chemical method for Nluc labeling, we performed the experiments, including labeling Nano luciferase to ch2C5 by maleimide method. We identified the LOD for this Nluc-ch2C5 by detecting serially diluted S-RBD protein by direct ELISA and double-antibody sandwich ELISA, the serially diluted concentrations of inactivated SARS-CoV-2 were also tested. The comparison of LODs among HRP-ch2C5, Nluc-ch2C5 by maleimide method(Nluc-MBS-ch2C5), Nluc-ch2C5 by genetic method(Nluc-ch2C5) demonstrated that Nluc-ch2C5 was more sensitive than Nluc-MBS-ch2C5, and Nluc-MBS-ch2C5 was more sensitivity than HRP-ch2C5 (Additional file 2: figure), suggesting that the genetic method is preferred for preparing the Nluc labeled antibodies, while for the antibodies whose sequences not obtainable, chemical labeling method can be used to prepare the Nluc Labeling antibodies.

To verify the utility of Nluc-ch2C5 in a chemiluminescence assay for detection of SARS-CoV-2 antigen, we used it to develop an AMCA. We optimized the AMCA by determining the best working concentration of the Nluc-ch2C5 (10 ng/ml), selecting the most efficient capture antibody (MW06) that recognizes variant strains, and determining the best incubation conditions to deliver the best results (data not shown). The evaluation results demonstrated that this AMCA could reach 100% specificity and 75% sensitivity for clinical samples, with the higher sensitivity than ELISA and colloid gold strip kits, showing the Nluc-ch2C5 works well in this AMCA.

However, in this study, we have only shown the effect of AMCA in detecting samples of SARS-CoV-2 original strain, this virus has been mutating since its emergence, from the Alpha, Beta, or Delta strain to the Omicron strain, and other variants may appear in the future. This AMCA can sensitively detect some of the variant strains (Alpha, Beta, and Delta strain cultures, data not shown), but might fail to cover all of the variants. To obtain a wider spectrum for more variants, specific antibodies against other variants may need to be supplemented and detecting availability should be evaluated further.

As a viral antigen test platform of this AMCA, the whole testing process completes automatically within 40 min, and the sealed cartridge can avoid contamination of the sample; thus, this automatic, safe, and very sensitive AMCA would be potentially useful in both local units and in less well-developed areas to detect SARS-CoV-2 infection at an early stage. Moreover, this AMCA can be used with different detection antibodies to detect different viruses, making it a very versatile assay.

## Conclusion

In this study, monoclonal antibody ch2C5 served as a model antibody, and the SARS-CoV-2 served as a model pathogen; we demonstrated that the Nluc labeled detecting antibody (Nluc-ch2C5) could significantly improve the detection sensitivity of SARS-CoV-2 antigen and could be utilized in AMCA. This labeling principle applies to other viral infections, so this labeling and test format could be expected to play an important role in detecting other virus antigens. In addition, combining the Nluc labeled antibody with immune chromatography technology and a small chemiluminescence detector will make it possible to develop a convenient, rapid, sensitive immune chromatography detection kit.

## Supplementary Information

**Additional file 1**. Test results for the positive SARS-CoV-2 clinical samples.

**Additional file 2: Figure**. LODs of Nluc-MBS-ch2C5. A: LOD of Nluc-MBS-ch2C5 for S-RBD in the direct ELISA. The S/C values comparing with HRP-ch2C5, Nluc-ch2C5 (left); LOD of Nluc-MBS-ch2C5 (right). B: LODs of Nluc-MBS-ch2C5 for S-RBD in the double-antibody sandwich assay. S/C values comparing with HRP-ch2C5, Nluc-ch2C5 (left); LOD of Nluc-MBS-ch2C5 (right). C: LODs of Nluc-MBS-ch2C5 for SARS-CoV-2 in the double-antibody sandwich assay. S/C values comparing with HRP-ch2C5, Nluc-ch2C5 (left); LOD of Nluc-MBS-ch2C5 (right). S/C is the value of the average luminance intensity for the test group divided by the cutoff value (average luminance intensity plus three standard deviations). The dotted line denotes S/C=1 (in left figures). The inner line is a Probit curve (does-response rule) and the outer dotted/dashed lines are the 95% confidence intervals (in right figures).

## Data Availability

Original data and materials are available from the corresponding author.
