# Supplementary material for: Preparation of the luciferase-labeled antibody for improving the detection sensitivity of viral antigen
Source: Virol J. 2022 Jul 28;19:126. doi: 10.1186/s12985-022-01855-6 (PMC9332066; doi:10.1186/s12985-022-01855-6)
Supplement: Supplementary file 1 — Additional file 1. Test results for the positive SARS-CoV-2 clinical samples. [file 12985_2022_1855_MOESM1_ESM.docx]

Supplemental table: Test results for the positive SARS-CoV-2 clinical samples.

| NO. | Nluc-AMCA | qPCR(CT) | ELISA | Colloidal Gold Test Strips |
| --- | --- | --- | --- | --- |
|  | （S/C） |  |  |  |
| 1 | 1.93 | 24.65 | **+** | **+** |
| 2 | 2.49 | 28.66 | **+** | **+** |
| 3 | 4.04 | 19.41 | **+** | **+** |
| 4 | 2.02 | 23.31 | **+** | **+** |
| 5 | 2.79 | 23.91 | + | **+** |
| 6 | 2.5 | 25 | + | **+** |
| 7 | 1.21 | 23.64 | + | **+** |
| 8 | 0.76 | 31.98 | - | - |
| 9 | 0.89 | 31.92 | - | - |
| 10 | 1.17 | 30.08 | - | - |
| 11 | 1.52 | 26.08 | - | **+** |
| 12 | 0.84 | 34.82 | - | - |
| 13 | 1.29 | 24.65 | **+** | **+** |
| 14 | 3.14 | 20.47 | **+** | **+** |
| 15 | 0.71 | 34.66 | - | - |
| 16 | 1.48 | 21.69 | + | **+** |
| 17 | 5.333696 | 19.99 | + | **+** |
| 18 | 2.334706 | 23.73 | + | **+** |
| 19 | 2.098913 | 24.39 | + | **+** |
| 20 | 1.561957 | 25.62 | + | **+** |
| 21 | 1.759259 | 30.34 | - | **-** |
| 22 | 2.28976 | 26.08 | - | **+** |
| 23 | 1.565998 | 24.65 | + | **+** |
| 24 | 14.66806 | 23.91 | + | **+** |
| 25 | 1.12694 | 25 | + | **+** |
| 26 | 1.290064 | 23.64 | + | **+** |
| 27 | 1.554898 | 26.08 | + | **+** |
| 28 | 1.077935 | 20.47 | + | **+** |
| 29 | 0.90729 | 31.53 | - | **-** |
| 30 | 0.503962 | 31.02 | - | **-** |
| 31 | 0.802848 | 34.88 | - | **-** |
| 32 | 0.70745 | 34.46 | - | **-** |

“+”,positive;”-“,negative. The S/C value higher than 1 is regarded as positive,all of the positive results byAMCA,ELISA and Colloidal Gold Test Strips are marked in red color.
