# Supplementary figures and images for: Preparation of the luciferase-labeled antibody for improving the detection sensitivity of viral antigen
Source: Virol J. 2022 Jul 28;19:126. doi: 10.1186/s12985-022-01855-6 (PMC9332066; doi:10.1186/s12985-022-01855-6)

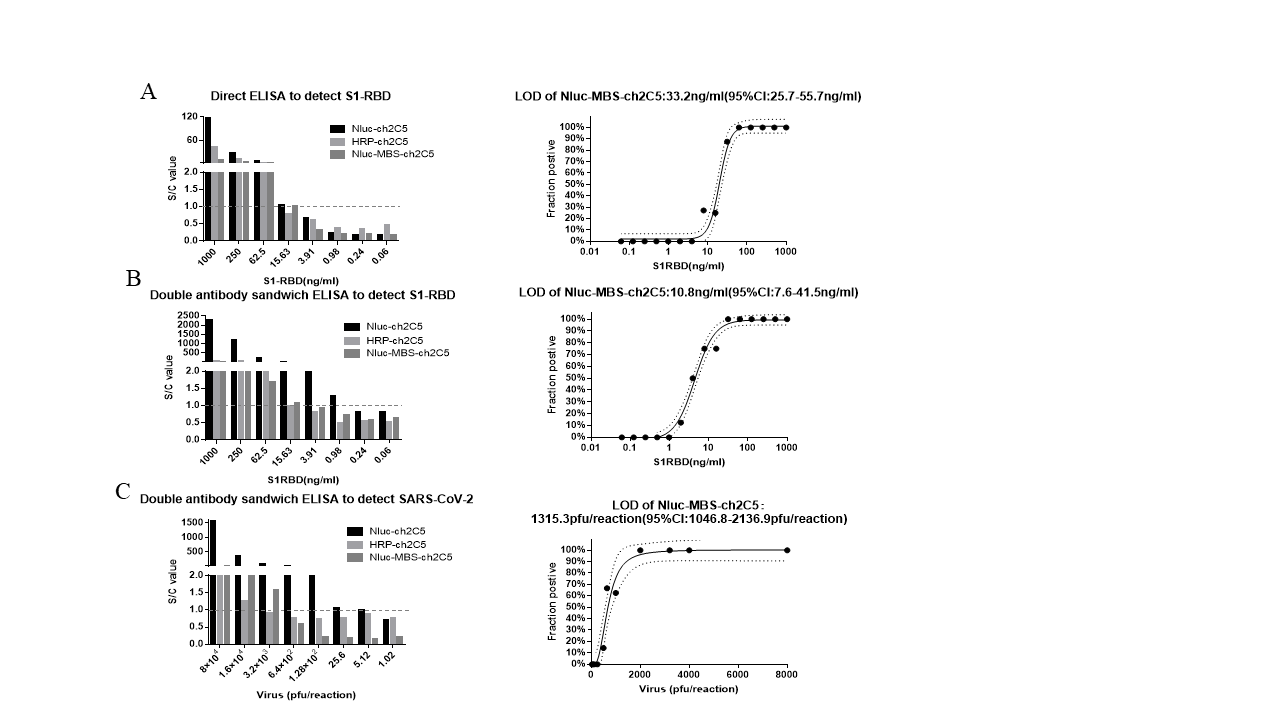

Supplement: Supplementary file 2 — Additional file 2: Figure. LODs of Nluc-MBS-ch2C5. A: LOD of Nluc-MBS-ch2C5 for S-RBD in the direct ELISA. The S/C values comparing with HRP-ch2C5, Nluc-ch2C5 (left); LOD of Nluc-MBS-ch2C5 (right). B: LODs of Nluc-MBS-ch2C5 for S-RBD in the double-antibody sandwich assay. S/C values comparing with HRP-ch2C5, Nluc-ch2C5 (left); LOD of Nluc-MBS-ch2C5 (right). C: LODs of Nluc-MBS-ch2C5 for SARS-CoV-2 in the double-antibody sandwich assay. S/C values comparing with HRP-ch2C5, Nluc-ch2C5 (left); LOD of Nluc-MBS-ch2C5 (right). S/C is the value of the average luminance intensity for the test group divided by the cutoff value (average luminance intensity plus three standard deviations). The dotted line denotes S/C=1 (in left figures). The inner line is a Probit curve (does-response rule) and the outer dotted/dashed lines are the 95% confidence intervals (in right figures). [file 12985_2022_1855_MOESM2_ESM.tif]
